# Supplementary material for: Analysis of CDPK1 targets identifies a trafficking adaptor complex that regulates microneme exocytosis in Toxoplasma
Source: bioRxiv. 2023 Jan 12:2023.01.11.523553. Preprint. [Version 2] doi: 10.1101/2023.01.11.523553 (PMC9882037; doi:10.1101/2023.01.11.523553)
Supplement: 10 [file NIHPP2023.01.11.523553v2-supplement-10.pdf]

bioRxiv preprint doi: https://doi.org/10.1101/2023.01.11.523553; this version posted January 12, 2023. The copyright holder for this preprint (which was not certified by peer review) is the author/funder, who has granted bioRxiv a license to display the preprint in perpetuity. It is made available under aCC-BY 4.0 International license.

Supplemental Figures

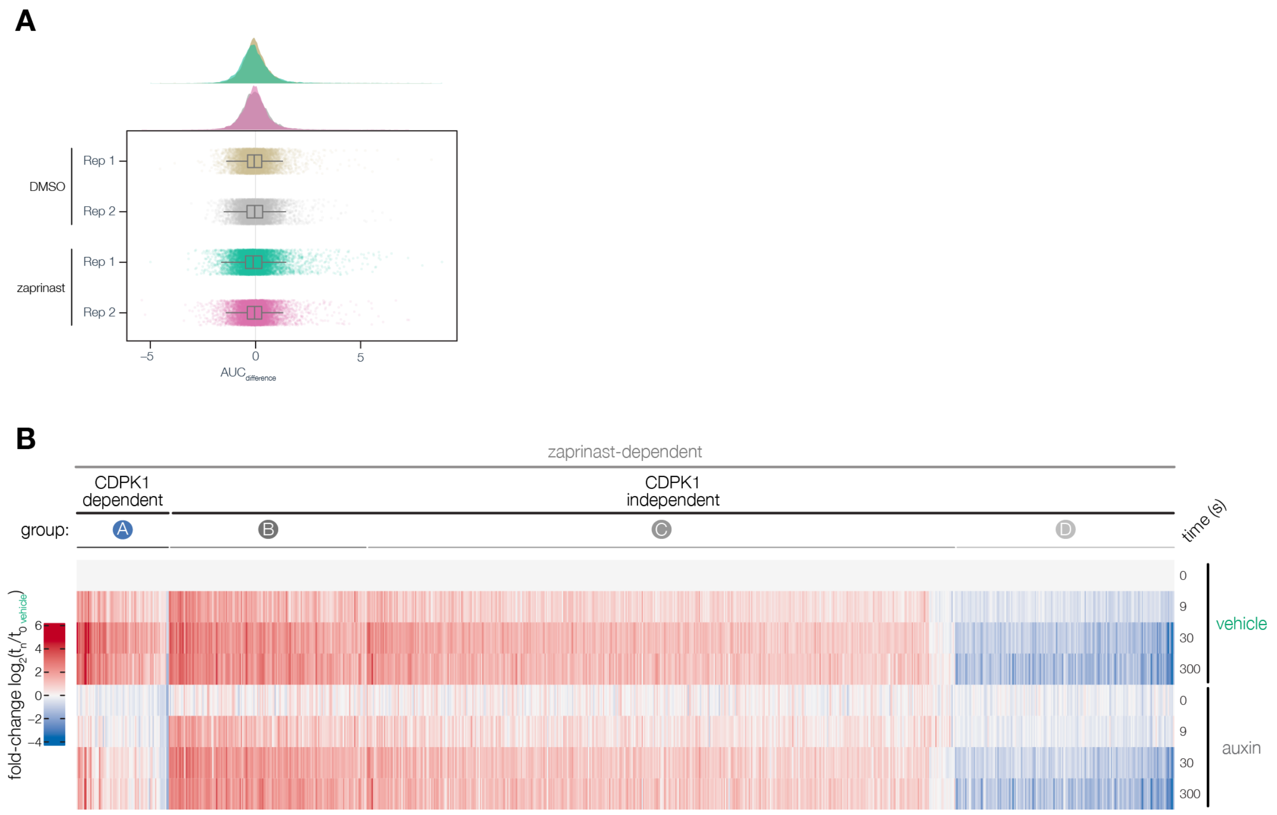

Figure S1. Zaprinast-dependent phosphoproteome.
(A) Distribution of $AUC_{difference}$ values for individual peptides in the enriched DMSO phosphoproteome and enriched zaprinast phosphoproteome across two biological replicates. **(B)** Heatmap of zaprinast-dependent phosphopeptide abundance ratios across time relative to the vehicle t0 interval during auxin or vehicle treatment. Peptides are organized by CDPK1-dependent phosphopeptides (Group A) and CDPK1-independent phosphopeptides (Group B, C, D).

bioRxiv preprint doi: https://doi.org/10.1101/2023.01.11.523553; this version posted January 12, 2023. The copyright holder for this preprint (which was not certified by peer review) is the author/funder, who has granted bioRxiv a license to display the preprint in perpetuity. It is made available under aCC-BY 4.0 International license.

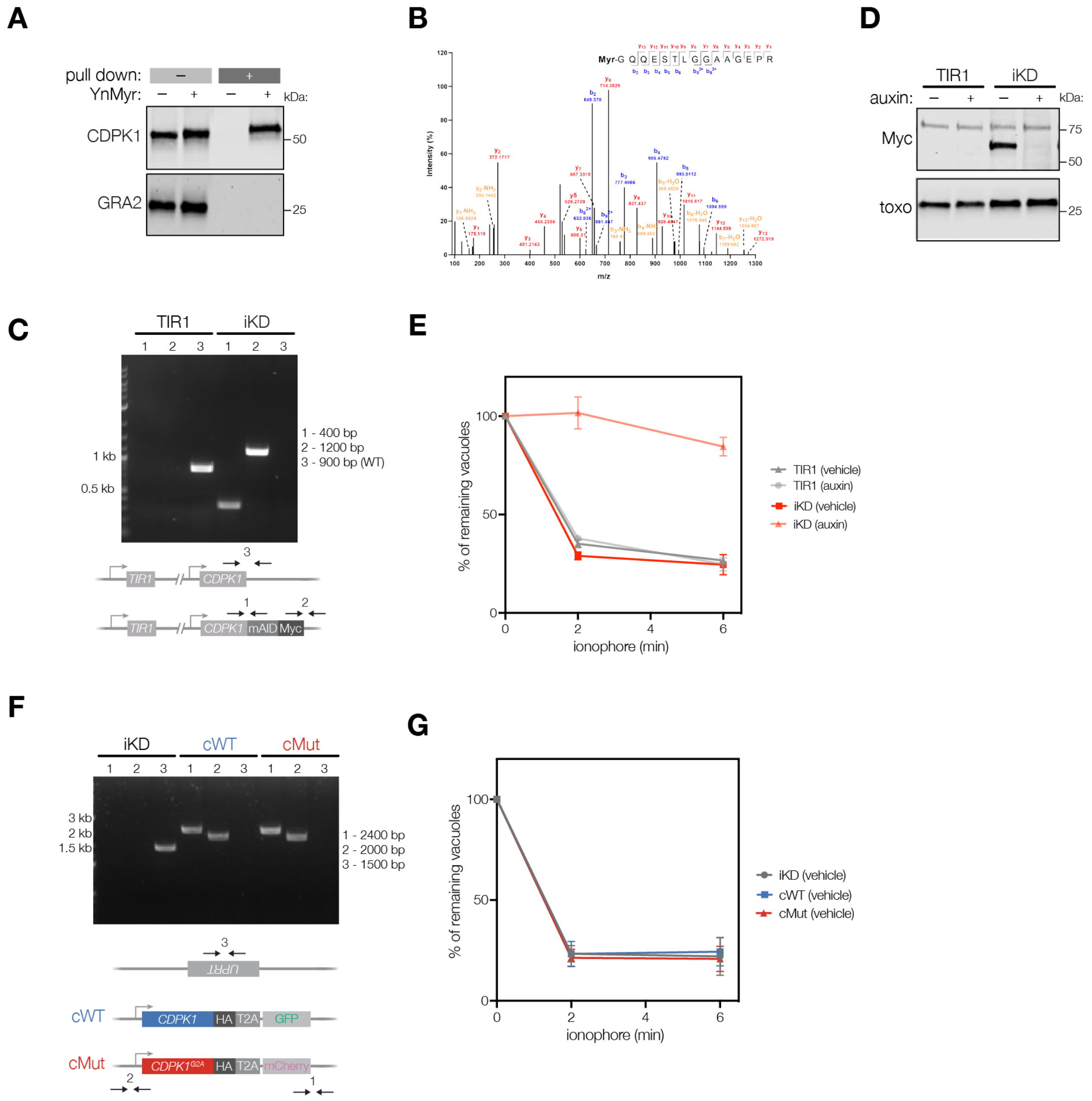

Figure S2. CDPK1 myristoylation, inducible knockdown, and complementation.
(A) YnMyr-dependent pull down confirming myristoylation of CDPK1. GRA2 antibody was used as a loading control. **(B)** MS2 fragmentation spectra indicating myristoylation of Gly2 of CDPK1 after YnMyr-dependent pull down. **(C)** PCR analysis confirming correct integration of the *mAID* cassette at the C terminus of endogenous *CDPK1* in the TIR1 line. **(D)** Immunoblot validation of auxin-dependent depletion of CDPK1 in the iKD line using the anti-Myc antibody and the anti-toxo antibody as a loading control. The band at 75 kDa represents anti-Myc-related background. **(E)** Conditional depletion of CDPK1 abolishes ionophore-induced egress from host cells. Intracellular parasites were treated with auxin or vehicle (EtOH) for 2 hrs and egress was initiated by addition of 8 µM A23187. The number of intact vacuoles was monitored over the course of 6 min. Each data point is an average of two biological replicates, each in technical triplicate, error bars represent standard deviation. **(F)** PCR analysis confirming correct integration of the complementation constructs encoding the WT (cWT) and myristoylation mutant (cMut) copies of CDPK1 at the *UPRT* locus of the iKD line. Primers are indicated by arrows. Base pairs (bp). **(G)** In the absence of auxin, both cWT and cMut parasites egress from host cells within 2 min post-ionophore treatment.

bioRxiv preprint doi: https://doi.org/10.1101/2023.01.11.523553; this version posted January 12, 2023. The copyright holder for this preprint (which was not certified by peer review) is the author/funder, who has granted bioRxiv a license to display the preprint in perpetuity. It is made available under aCC-BY 4.0 International license.

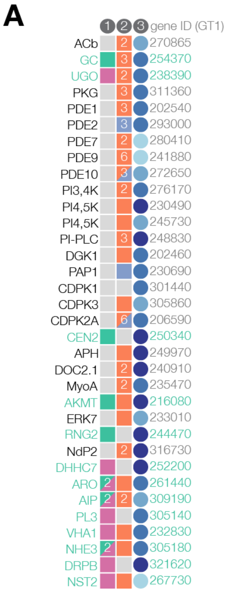

Figure S3. Factors controlling parasite motility.
(A) Expanded list of factors involved in parasite motile stages from Figure. 3H.

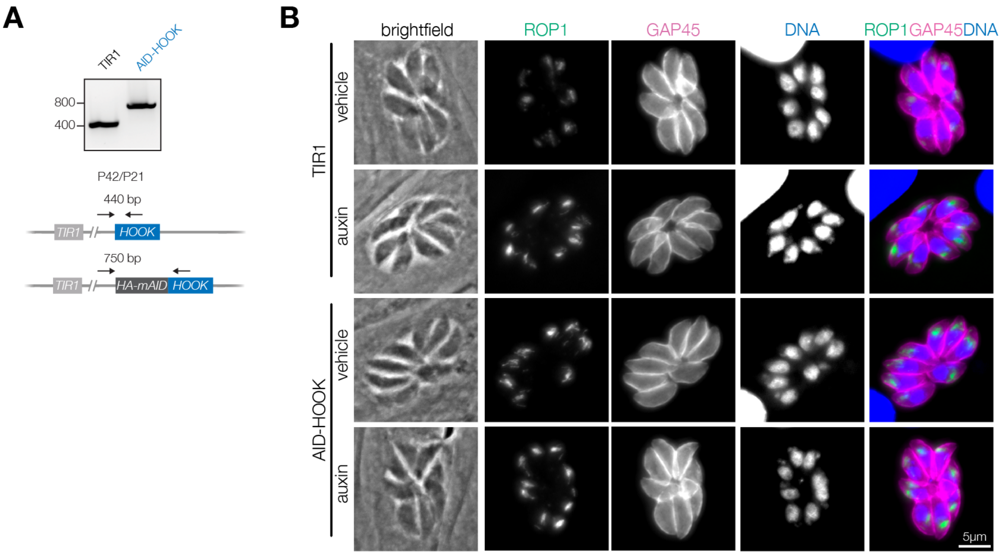

Figure S4. Extended analysis of HOOK knockdown.
(A) PCR analysis confirming correct integration of the *HA-mAID* cassette at the N terminus of endogenous *HOOK* (*TGGT1_-289100*) in the TIR1 line. **(B)** Rhoptries (ROP1) are visualized in fixed intracellular parasites by immunofluorescence after treatment with auxin for 24 hrs. Hoechst and GAP45 are used as counterstains.

bioRxiv preprint doi: https://doi.org/10.1101/2023.01.11.523553; this version posted January 12, 2023. The copyright holder for this preprint (which was not certified by peer review) is the author/funder, who has granted bioRxiv a license to display the preprint in perpetuity. It is made available under aCC-BY 4.0 International license.

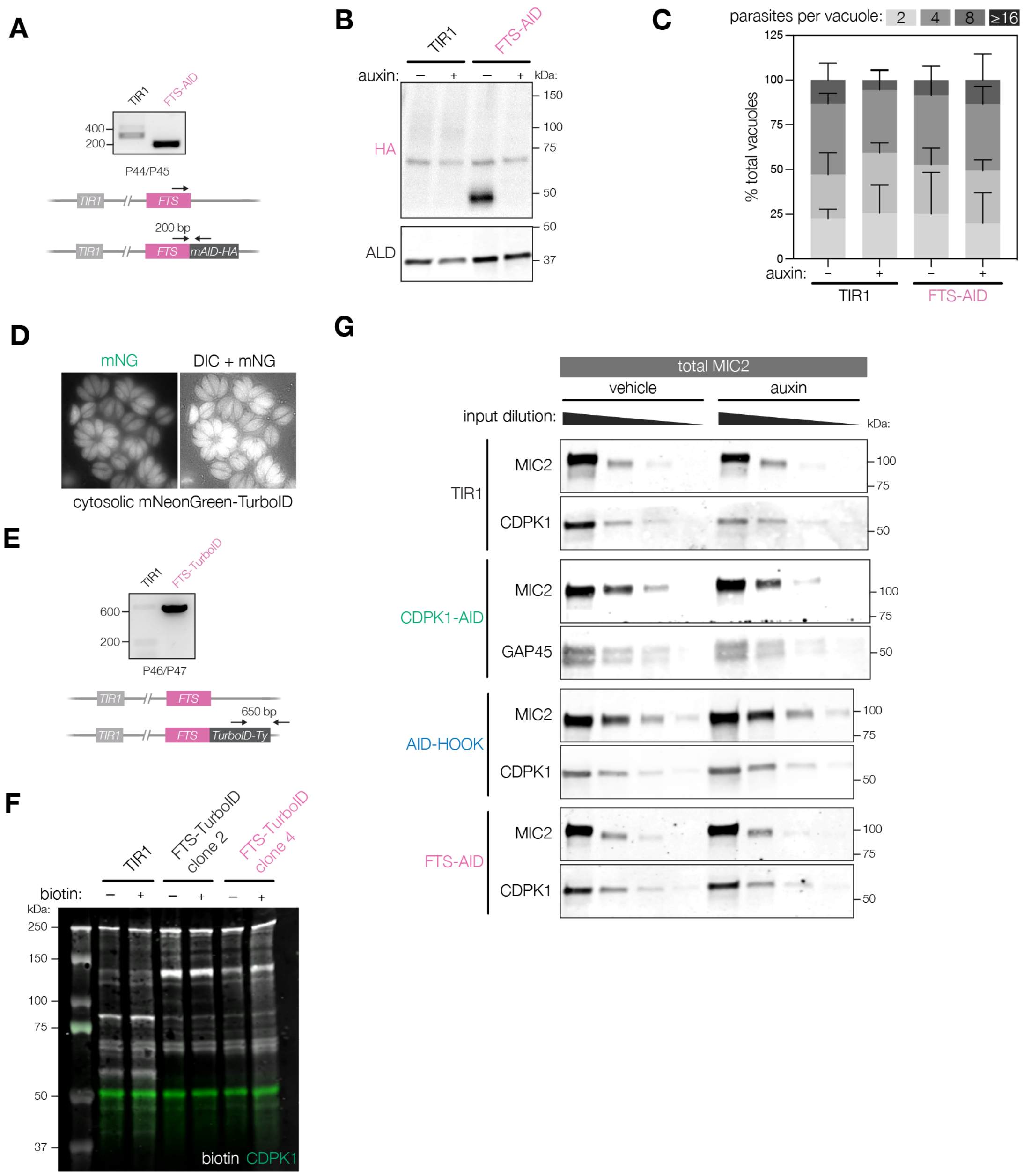

Figure S5. Extended analysis of FTS knockdown, proximity labeling, and microneme protein secretion.
(A) PCR analysis confirming correct integration of the *mAID-HA* cassette at the C terminus of endogenous FTS (*TGGT1_264050*) in the TIR1 line. **(B)** Uncropped immunoblot shown in Figure 5D confirming C-terminal tagging of FTS. The band at 70 kDa represents anti-HA/anti-ALD-related background present in all conditions. **(C)** Replication assays of host cells infected with TIR1 or FTS-AID parasites in auxin for 24 hrs. Parasites per vacuole were quantified from immunofluorescence on fixed intracellular parasites. $p > 0.9$. Two-way ANOVA. **(D)** Live microscopy of HFFs infected with parasites expressing cytosolic mNeonGreen-TurboID as the cytosolic control for proximity labeling. **(E)** PCR analysis confirming presence of *TurboID-Ty* cassette in the TIR1 line. **(F)** Immunoblot detection of biotinylated proteins in FTS-TurboID and cytosolic mNG-TurboID parasites treated with 500 µM of biotin or a vehicle of DMSO. Biotinylated proteins detected with a labeled streptavidin. anti-CDPK1 antibody was used as a loading control. **(G)** Serial dilution of total parasite lysate for cKD strains for TIR1, CDPK1, HOOK, and FTS used in microneme protein secretion assays in Figure 5J to generate standard curves.

bioRxiv preprint doi: https://doi.org/10.1101/2023.01.11.523553; this version posted January 12, 2023. The copyright holder for this preprint (which was not certified by peer review) is the author/funder, who has granted bioRxiv a license to display the preprint in perpetuity. It is made available under aCC-BY 4.0 International license.

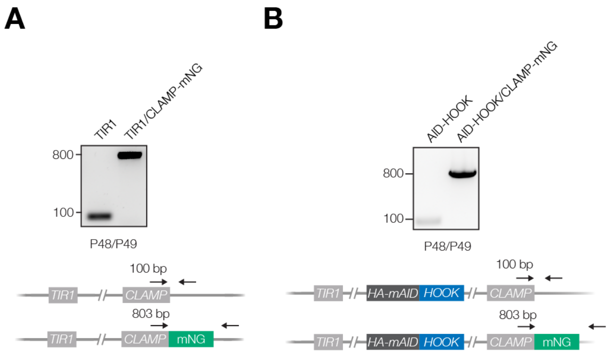

Figure S6. Extended analysis of FTS knockdown, proximity labeling, and microneme protein secretion.
(A) PCR analysis confirming correct integration of the *mNeonGreen* reporter at the C terminus of endogenous *CLAMP* (*TGGT1_-265790*) in the TIR1 line. **(B)** PCR analysis confirming correct integration of the mNeonGreen reporter at the C terminus of endogenous *CLAMP* in the AID-HOOK line.
